# Abstract of the Proceedings of the Buffalo Medical Association

**Published:** 1858-01

**Authors:** Austin W. Nichols


ART. V.—*Abstract of the Proceedings of the Buffalo Medical Association.*

TUESDAY EVENING, Dec. 1, 1857.

The association met at the usual hour.

Present—The President, Dr. Flint, in the chair. Drs. Wyckoff, Newman, Lemon, Gay, Root, A. Flint, Jr., Eastman, Rogers, Hawley, Gould, Wilcox, Rochester, Hamilton, Miner, Hutchins, Ring, and A. W. Nichols.

The secretary being absent, the reading of the minutes of the last meeting, on motion, was dispensed with.

Dr. A. W. Nichols was appointed secretary pro. tem.

*Dr. Miner*, chairman of the committee on Dissection Wounds, then read an interesting and elaborate report on dissection wounds, quoting a number of cases in support of his arguments, which we regret cannot be published in the Journal, though they are of great interest, as the space will not permit. The following is an abstract of Dr. M.'s report:

*Report on Dissection Wounds.* By J. F. MINER, M. D.

In response to the invitation of the association, and in obedience to my own feelings of interest, I have somewhat industriously searched for the reported cases of post-mortem poisoning, which, together with those furnished by my friends, and those which have fallen under my own observation, I wish very briefly to present, prefacing the facts by the simple remark, that upon the same testimony and quoting the same cases, equally distinguished observers have been led to the most opposite and conflicting opinions.*

Case I. Jan. 5, 1850, my brother, D. N. Miner, M. D., of Ware, Mass., received a very slight prick upon the middle finger in sewing the body of a child dead fifteen hours of acute peritonitis. The following day was attacked by pain in the head, back, and bones generally. Some rigor, thirst, heat, and great nervous irritability. Fever of a low, typhoid character, continued for some time, during which the state of exhaustion was extreme; to speak, move, or even breathe, seemed too much effort for his exhausted nervous power.

In about two weeks abscesses formed upon, and under the muscles of the shoulder, chest, and arm, which when opened discharged quantities of healthy looking pus.

Abscesses continued to form for ten or twelve weeks, greatly reducing his strength and producing extreme emaciation. The local effects at the place of puncture were very slight, not appearing at all for three weeks, and then only a small pustule, around the base of the nail, the nail gradually separating from its attachments.

Treatment consisted in poultices on the abscesses, and early opening, which even as soon as detected would not unfrequently discharge half a pint of pure pus. Constitutionally: brandy, quinine, opium and customary diet.

Under this treatment he gradually regained his health after a year's illness.

Case II. During the illness of my brother, while the matter from the abscesses was discharging so freely, my mother, 64 years of age, robust and

---

* The following are some of the references to cases, made by Dr. Miner:
New York Journal, vol. ix, p. 209.
North American Med. Journal, vol. v, p. 106.
Good's Study of Medicine.
American Journal, vol, i, p. 315.
Medical Repository, No. 113.
Mr. Travers on Hoisoned Wounds, Med. Chirurg. Review.
Med. News and Library, vol. xv, No. 175, p. 110.
Lancet, May 16, 1857.

healthy, at 4 or 5 o'clock, P. M., made a slight scratch upon her thumb with a pin while washing some of the linen which had been worn upon the person of her son.

She passed a restless, sleepless night; suffered agonizing pain in the thumb and hand; severe chill; high fever; great thirst.

The most positive signs of mortification were presented in the short space of eighteen or twenty hours from receipt of injury.

In twenty-four hours the hand and arm was frighfully distended. Pain now increased to agony; large mortified patches upon the hand and forearm; inflammation extending to the tissues of the shoulder, neck and chest, terminating in mortification of the whole arm and shoulder previous to death. Died comatose on the fourth day.

*Treatment.* Brandy, opium, poultices.

I wish, also, in this connection, to read a brief account of my own case, though it seems quite unnecessary, as many members of the society kindly visited me during my illness, besides Dr. Root, Prof. Flint, and Prof. Hamilton, to whom I am vastly indebted for attendance. Still as it is the third case in the same family, and may have some bearing upon the subject of constitutional peculiarity or susceptibility, I shall be pardoned for reading it.

Case III. March 21, 1857, made post-mortem examination of a child dead some fifteen hours of pleurisy, with effusion, left lung being compressed and that side of chest filled with serum and pus. In sewing up the body the eye of the needle passed apparently into the scarf skin only, upon the end of the thumb.

Eighteen hours after experienced pain in the thumb, soreness of the axillary gland, and by shaving off the outer skin found a small collection of a milky fluid at the point of puncture.

In twenty-four hours whole hand and arm excessively painful, with considerable redness of the forearm and swelling of the axillary glands. System affected with high irritative fever.

In thirty-six hours thumb opened to the bone, which at the end was completely mortified; very little matter escaped.

On the third day signs of mortification extending, Prof. Hamilton made a deep lengthened incision through distended thumb. The whole hand seemed nearly as much involved in the inflammation as the thumb.

The inflammatory action gradually abated after about a week. The end of the thumb involved in mortification, at length separated, bringing with it some small pieces of bone.

One abscess formed over the ulna, which was early opened, and rapidly healed.

At this time, six months after injury, the whole hand enlarged and stiffened.

Treatment consisted of yeast poultices to the hand and arm; opium to allay the agony of pain; quinine, brandy, champaigne and other tonics and stimulants.

Wm. Lawrence says in his lectures before his class, published in Medical Gazette, copied from Medico-Chirurgical Review of 1829–30:

"It seems to me very doubtful in these cases, whether any thing that can be called *virulent* or *poisonous* is introduced into the human frame by these occurrences, or whether these effects are to be explained as resulting from such injuries considered mechanically.

"As to the constitutional disturbance that may ensue in conjunction with these local symptoms, it is of an inflammatory character and must be treated by antiphlogistic means, according to the extent of the disturbance. I acknowledge I am inclined to discourage as much as I can the idea of a poisonous nature attached to these wounds, because I conceive the opinion produces much alarm. I do not, however, argue against their poisonous nature from this motive, but I give you my opinion formed from the consideration of the phenomena independent of any view of that kind.

"I am certainly glad that I arrive at this conclusion, any other opinion would lead you to prosecute your anatomical studies with greater anxiety."

He then quotes cases illustrative of these views, which are here omitted.

Mr. Benjamin Travers, in his work on Constitutional Irritation, seems to have followed a careful line of observation. He divides his cases into simple injuries, and absorption of specific poison. He proves by a case, that inflammation is not necessary to the most virulent and fatal action of the poison.

As cases of simple irritation he also speaks of an unhealthy cook who was attacked with pain, swelling, fever, &c., &c., after boning a stale hare. Yet he says:

"It is probable that the irritation in some of these cases, though strictly local, is in its nature such as to act upon the constitution with greater severity than that of similar clean wounds."

Thus it will be seen Mr. Travers has narrowed down his position and almost abandoned his class, the result of simple irritation; for the other, named absorption of poisonous matter; or if not the absorption, at least the peculiar action or irritation of such matter.

Mr. Travers gives: "Milky vesicle upon the site of the wound; intense pain in the shoulder; erythematous inflammation, together with extreme dejection of spirits and constitutional suffering, as truly diagnostic of a case of absorption: while a simple local irritant is both liable and competent to produce erysipelatous redness of the wounded hand, pain passing up the arm; enlarged glands and deep-seated abscess in the axilla, with symptomatic fever of suppuration."

That there is a specific poison generated in human, and some animal bodies, *soon* after death, modified by, but not limited to, any particular forms of disease, existing very soon after death and rendered inert or less virulent by time or the process of putrifaction, we may consider pretty well established. While as nothing definite or satisfactory is known of the nature or character of this agent, and I avoid a mention of all speculation concerning it, yet I will suggest that whatever may be its properties, it seems capable of transmission through one victim to another undiminished in its strength, perhaps unchanged in its character, certainly unchecked in its fatality, though perhaps more facts and experiments are wanting to fully justify the conclusion.

In evidence of this I will refer you to the case of my brother and mother, which are, I conceive, sufficient to establish my position; there are numerous other cases on record pointing to this same fact, which are here omitted for want of space.

That poison exists, or is generated in all dead bodies, we have no means of certain knowledge. It seems probable, however, that it does exist in a greater or less degree.* Yet it will by no means follow that anatomists uniformly suffer. In the dissecting room the great majority of subjects are comparatively old. The plao of chemical affinities, which have been supposed to create during their action some new and poisonous compound, has subsided, in a great measure, if it ever existed. Again, the medication to which these bodies are subjected by alcoholic baths and injections of kreosote, arsenic, &c., into the vessels to preserve them from decay, may reasonably be supposed to produce a counteracting effect.

It is the generally expressed opinion, and appears from all the facts I am

---

* Garcilaso de la Vega, in his History of the Spaniards in the Indies, part ii, vol. i, chapter 42, observes: "All the Indians in the westward islands poison their arrows by dipping their points in dead bodies."—*Good's Stuby of Medicine.*

able to collect, that *time* also, has an important influence, connected as it is with heat, cold, moisture, and other atmospheric conditions.

Most cases of poisoning arise from dissections made very soon after death. Hence, though a body possess the poisoning quality in a feeble or imperceptible degree a few days later, it may not be inferred that previously it did not contain the most concentrated and potent poison.

Yet after all these protecting and controlling influences, together with the usual good health, and correct living, of medical students and anatomists, still wounds received, are often, indeed I think *usually*, attended by an unnatural irritation and an uncommon reluctance in healing. Also by more tingling and smarting pain than is usual from other injuries, if fortunately unattended by no more serious consequences. Mode of introduction, constitutional peculiarity or susceptibility, general health and habits of life, go far to determine the question of risk. An open, free bleeding wound, seems quite unattended with danger, compared with the slight pricks which may be scarcely seen or felt.

According to Liston, "inocculation is dangerous when the body of *any* person has become putrid; also when the body is recently dead, especially if it has been a case of puerperal disease. Symptoms are most violent from those wounds."

"The contagious material seems also to pervade alike all the fluids of the decomposing body, not being at all confined to the morbid parts." The disease follows where the abraded surface comes in contact with fluids from the stomach, secretions from serous or mucous membranes; when it has touched a towel or sponge used in wiping up the fluids or other material requiring to be removed. Dr. Cumming only threaded a needle which had been wetted. It was followed by fatal poisoning.

Again, the existence or character of the contagious material, would seem in no very exclusive manner to depend upon the disease of which the subject has died. It has been held to follow only after inflammation of serous membranes, puerperal disease producing it with the greatest virulence.

The cases collected, though wholly inadequate to establish the proportions by different diseases, still show that it is not confined to any particular one. No attempt has been made to collect a great number. Of the few obtained we find

| | | | | |
|---|---|---|---|---|
| From subjects dead of | Peritonitis, | 4, | Fatal, | 2 |
| " " " | Pleurisy, | 1, | " | 0 |
| " " " | Puerperal Fever, | 4, | " | 3 |

| | | | | |
|---|---|---|---|---|
| From subjects dead of | Hæmorrhage, | 2, | Fatal, | 2 |
| " " " | Consumption, | 2, | " | 2 |
| " " " | Enteritis and Erysipelas, | 1, | " | 1 |
| " " " | Typhus, | 1, | " | 1 |
| " " " | Croup, | 1, | " | 0 |
| " " " | Unknown, | 4, | " | 2 |
| | Whole number, | 20 | Fatal, | 13 |

Reference is also made to poisoning received

| | | | |
|---|---|---|---|
| From dead animals, . . . . | 3, | Fatal, | 2 |
| Communicated to attendants, . . . | 9, | " | 3 |

There seems to exist some curious relationship between post-mortem poisoning and erythematons or erysipelatous inflammation. Dr. Good, in his Study of Medicine, has treated it under the head of erythema anatomicum.

Dr. Ebenezer B. Pulling, assisted in the examination of a woman who died of hæmorrhage. In the course of a few days he complained of pain in a finger which he had wounded. From a point in the finger, the swelling extended to the middle of the arm above the elbow, and was there arrested. Soon after, swelling commenced on the same finger of the other hand, and spread thence almost over the whole body. It proved fatal, and this case was the beginning of a fatal epidemic of erysiplas, the first case of which was a clergyman, who had acted as nurse to Dr. Pulling.

Cases might be introduced indicating the conveyance of erysipelatous disease to lying-in-women, and also the virus of dead animal bodies, which might be conveyed, even though much care had been taken, if soon brought in contact with the delicate surfaces of the vagina and neck of the uterus.

This would show the absolute criminality of physicians making post-mortem examinations, and immediately, or soon after, attending obstetric practice; also the prudence of declining obstetric practice during attendance upon erysipelatous disease of any kind. I do not regard this the place to introduce them, yet cannot refrain making the above practical suggestion, leaving the further consideration of this branch of my subject for some future occasion.

It may be observed that in most of these cases, the brain and nervous system sympathise quickly and deeply. They mostly exhibit strong excitement in the commencement, and early and rapid prostration. The stomach and diaphragm are affected in some, not in others. Local inflammation, stronger in some than others. In some there is a marked affection of the absorbent vessels, with erythematous, serous or suppurative inflammation of the cellular substance of the arm, axilla, and breast. Mortification seems in some cases to be produced without the prelimlnary stage of inflammation, so virulent and fatal is the action of the poison.

These symptoms, then, are to be met by appropriate treatment. I have some delicacy in attempting to indicate what may be appropriate when so great differences exist in the opinions of the most eminent authorities. I have already indicated the views of many of the most distinguished physicians and surgeons, both of our own and foreign countries, in reporting the cases with which they have been connected.*

Sir Astley Cooper, in his eighth lecture on poisoned wounds, has given in a condensed form, his views of the nature and treatment of dissection wounds.

"I attribute the bad effects occasionally taking place in dissection wounds, not to any morbific matter introduced into the wound, but to the irritability of the person's constitution at the time. From imagining that a putrid diathesis existed, many persons were in the habit of giving wine and bark, and never allowed that the symptoms resulted from inflammation; the consequence was, many died under this plan of treatment."

Sir Astley himself, some years since, suffered from such accident. He called a friend, who prescribed wine and bark. He submitted to this for some time, during which all the symptoms became aggravated. He then changed the treatment to antiphlogistic measures and got well. He recommends caustics, nit. acid if the wound be deep; leeches and fomentations; antiphlogistic regimen adopted to its *full extent;* calomel succeeded, or accompanied by saline purgatives; opium and antimony according to the discretion of the surgeon.

I should be happy to quote more fully the opinions and practice of physicians, but will say only they seem divided: some maintaining and adopting one extreme, some the other. I will briefly mention some of the means used to prevent the absorption of poison after wounds have been received.

Some interesting experiments by M. Bouillard, published in the Archives Générals du Médecine, Nov., 1826, would seem to show a value in ligaturing immediately above the wound, and then by washing, suction by mouth or cupping glass, removing as much as possible, the virus.

Caustic and other applications to the wound are generally and with good reason adopted. Nit. acid, nit. argenti, tinct. iodine, &c.

---

* Many of these cases re omitted for want of space.—Ed. Jour.

To nutralize it when once introduced, or with the view to prevent its poisonous action, mercury and iodine have been suggested and used, but with no positive results to justify its employment. Mercury has also been employed on account of its supposed control over inflammatory action.

The depressed and agitated nervous system, which in almost all cases is violently affected, should certainly be soothed and quieted. The local or general pain which often amounts to an agony, should most certainly be relieved and moderated as much as possible. Opium then, in some of its forms, is urgently demanded in the treatment of most, if not all the cases of post-mortem poisoning.

In these cases, there is generally great depression, with early and rapidly increasing prostration. This can most effectively be met by stimulants and tonics—brandy and quinine, early and late, *first* and *last*. And by this I mean most of these cases will very early admit and demand support; later in the disease it will form our chief reliance.

The high irritative fever and the severe general and local pains, have been treated by free and repeated venesections. This has been quite generally adopted and is sustained by the highest and most commanding authority. Most of the reported fatal cases have been bled; I had almost said all the severe cases which have been largely bled, have been fatal. I think I have presented to you some remarkable illustrations of the power of nature to overcome both disease and treatment combined, a case, where from three to four quarts of blood was drawn from the arm, and two or three hundred leeches applied.

While the system generally is sustained, it may sometimes be necessary and proper, locally to deplete; and I wish earnestly to indorse and recommend the plan adopted by several surgeons, of freely and deeply laying open with the sealpel highly inflamed, distended, tense, and painful parts. A favorable result in many cases will depend mainly upon this plan being early and fearlessly adopted. There is no inconsistency in sustaining the system on the one hand, and endeavoring to overcome local inflammation by incisions, cupping, leeching, &c., on the other.

I have attempted nothing new, or designed attacking anything old; avoided theories of my own and others, and condensed the reports of cases as mueh as consistent; yet an apology is due you for trespassing upon your time so long, and also for presenting this subject in so brief and imperfect a manner, leaving so much unsaid, so many cases uncollected, and so many opinions of accurate observers unnoticed.

*Prof. Hamilton* remarked that he should but respond to the opinion of members of the society, when he said that they had listened to an able and interesting paper from Dr. Miner. The fact, however, that it was an able paper, rendered it the more necessary that just criticisms should not be omitted. It contained in his judgment, one blemish which ought not to pass unnoticed. The writer believed all dead bodies generated a poison, and presented in evidence of this fact a traveler's tale that a certain tribe of Indians dipped their arrows in dead bodies before entering battle. Dr. Hamilton did not believe that any tribe of Indians ever practised such a method of seeking the death of their enemies. The statement was wholly improbable, and unworthy of a place in such a paper. And if this was all the evidence upon which the writer's belief in the universal generation of poison in dead bodies, rested, then it was inconclusive.

Dr. Hamilton was unwilling that such an opinion should receive the sanction of this society. There were present a great many young men who would be afraid to prosecute their dissections; but he would remind them that we, who are their seniors, have dissected a great deal, and often been cut and scratched, and yet that we have never been poisoned. It is an exceedingly rare event that a student is poisoned in the dissecting-room. In his opinion this poison was not generated constantly in dead bodies, but only under peculiar circumstances, having relation to the character of the malady occasioning death, to the tissue diseased, and probably also to the peculiar agency of air and moisture after death, in consequence of which certain chemical changes were wrought which we do not yet understand.

Dr. Hamilton referred also the testimony of Parent Duchatelet, in relation to the general healthfulness of those employed constantly in large dissecting rooms, and to the condition of health observed in the women and children employed at the Chantieres d'eguarrissage, Paris. He had himself seen them sitting in piles of putrid meat picking the flesh from the bones; and in this occupation they must wound themselves often, still they look healthy, except as they indicate the influence of sedentary habits and confinement.

This Dr. Hamilton only presented as a kind of collateral testimony upon the point on which he was speaking, but it had its value.

*Dr. Miner* replied that Dr. Hamilton had entered the Hall late, and had not heard the remarks which preceded and accompanied the statement upon which his animadversions were based; that the paper did not make any very positive statement concerning the existence of poison in all dead bodies, and distinctly avowed that we had no means of certain knowledge. Yet from indirect and collateral evidence, such as he had presented he was of opinion that poisonous properties did exist in all dead human bodies, for a longer or a shorter period, in greater or less degree, modified by influences which we do not understand. Garcelaco de la Vega's account was very likely chimerical, he had not introduced it as evidence, or given it a place in the body of the paper; had merely referred to it, giving credit to Good's Study of Medicine, as the place from which it was obtained. It was referred to only as a story, expecting it would be received as traditional, and not by any means as conclusive proof or reliable medical testimony; he hoped the paper would give the medical students present no unnecessary anxiety, since Prof. Hamilton is of opinion poison exists only *very rarely*, and Wm. Lawrence, before his class, was *very glad* to think it did not exist *at all.*

*Prof. Hamilton* replied that with the explanations made by Dr. Miner, he was satisfied that his criticisms were unjust, but he regretted that Dr. Miner should have seen fit even to mention so doubtful a story as that of the Indians; it would be very likely to strike others as it did Dr. H., as insufficient testimony.

*Prof. Rochester* remarked that in furtherance of Dr. Hamilton's suggestion, as the paper now legitimately belong to the society, he would move that the paragraph containing the account by Garcelaco de la Vega, be stricken from the paper, if Dr. Miner had no objection.

*Dr. Miner* did not desire to make any defence for the paper or any part of it; was willing the society should do with it as it saw fit; he did not consider Dr. Hamilton's criticisms in any way unjust or unfair, and would not like to have them withdrawn. If Dr. Hamilton could sustain his position it was desirable he should do so. Dr. Hamilton had referred to the testimony of Parent Duchatelet which Dr. Miner considered as wholly opposed by universal and well known facts. That large dissecting rooms and all places where vapor from decaying animal matter was largely and constantly inhaled were uniformly unhealthy; that diarrhœa and constitutional disturbance, with loss of appetite, was a very common and almost constant consequence; that *this* account was not unlike the one opposed; to reject the first, and embrace the last, was only refusing the account of the historian and adopting known error.

*Dr. Root* then reported the following case:

*Cartilaginous Degeneration of Mitral Valves and Thickening of the Aortic Valves.*

Thomas Grady, aged 45 years, tinner, Irish.

I was first called to see Grady, on 28th Oct. last. He stated that he had been ill more or less for the past seven years, though for the greater part of the time he had been able to work at his trade, that of a tinner. He had been treated for consumption and dropsy of the heart, and had taken much medicine. He had the impression that he had consumption. For the last four years he had at times attacks of hæmorrhage from the lungs; difficult breathing; cough, which was pretty constant, and night sweats occasionally. When I first saw him he had just returned from the east, where he had been in search of work, and while in Rochester had an attack of hæmoptysis and difficult breathing.

*Present Symptoms.* General aspect pallid and emaciated to a moderate extent, though he always was rather thin; sitting in his chair; breathing labored; cough; expectoration of frothy mucus; no hæmoptysis; vomiting occasionally. His chief complaint was of a pain in the right groin, the seat of an old hernia. The pulse was full, strong, and about 80; respirations not enumerated. Percussions, in different regions of the chest, developed nothing unusual; I did not discover greater or more extended dullness over the præcordial region than normal; vesicular murmur intense, and mucous râle before a paroxysm of cough and expectoration. Sounds of heart over præcordial region loud and impulse strong. I did not detect any valvular murmurs.

I placed him on a tonic and sustaining course of treatment, and in a few days he was out of doors.

Nov. 16, 9, P. M., was called to see Grady. Had been at work that day at his trade, and had made three boilers in a little over a half day; he came home very much exhausted. Respiration very labored; cough, with expectoration of mucus mixed with blood; had vomited several times; pulse *full, strong* and *labored*; was sitting up in his chair but was very restless. The asthmatic symptoms and restlessness continued to increase till 6, A. M., when he died. The autopsy was made twenty-seven hours after death by Dr. A. W. Nichols.

*Post-mortem* examination confined to cavity of thorax.

The pleura was adherent on both sides, by recent and ancient exudation. The lungs were considerably congested, the right to a much greater extent than the left.

There were no signs of pericarditis; the amount of fluid in the pericardial sac was slight.

The heart was not much increased in size, being 4½ inches long and 4¼ wide. It weighed 17½ ounces. The ventricles were hypertrophied: the right ventricle was half an inch in thickness, and the left sixth-eighths of an inch. The right ventricular cavity was not enlarged. The left ventricular cavity was smaller than natural.

The aortic valves were contracted and thickened. The mitral valves were extensively diseased, being thickened, contracted, puckered, and filled with large deposit of calcareous matter.

The cause of death in this patient is thus apparent: congestion of the lungs from obstruction of mitral valve. The heart is seen to be moderately hypertrophied and of the kind called *concentric*. The ventricles were of less capacity than normal—particularly the left. The chief point of interest to me is the fact of the pulse being uniformly, as far as I could discover, *strong* and *full*. The strength of the pulse is explained by the existence of hypertrophy, which was the diagnosis of the case. The hypertrophy was suspected from the strength of the pulse and impulse of the heart; usually in obstruction of the mitral valve wi.h hypertrophy, the pulse is said to be *small* and *not well developed*, but were it was strong and full. The fullness was owing to the small capacity of the left ventricle. The mitral obstruction was not so great but that enough blood could be forced through to fill the whole of the ventricle, and the ventricle being full and *hypertrophied*, on its contraction, the full and strong pulse necessarily results.

From the nature of the case, the valvular murmurs, if any existed, were not easily detected. The mitral orifice was so small and of such shape, that regurgitation could not take place. Whether there was aortic regurgitation I am unable to say: none was detected. The hæmoptysis was of course owing to the congestion of the lungs, and the congestion arose from the mitral obstruction.

The fullness of the pulmonary artery, left side of the heart and venæ cavæ, was caused by the same obstruction of the valve acting through the capillaries of the lungs.

*Dr. Wyckoff* reported another case of heart-disease, as follows:

Oscar A. Hall, aged 9 years, called at my office, in company with his mother, Oct. 8, 1857, complaining of great weakness and inability to make any exertion; said that he could not walk fast or far without stopping to rest; had constant palpitation of the heart, which was distressingly increased by exercise; had also at the time some diarrhœa; eyes looked yellow; countenance pale and rather tumid; pulse frequent and feeble. Upon making a physical examination found evident signes of hypertrophy, also a very feeble but *distinct bellows murmur:* the second sound of the heart was very feeble and indistinct.

Prescribed for the diarrhœa, expecting to see him again very soon: did not see him again, however, until Nov. 3d, when I was sent for to see him; found him suffering very much from dyspnœa, also a slight cough, rather dry; unable to lie down; face palid and swollen; feet and ankles also swollen; pulse thread-like, and 160 per minute. Upon physical examination, found no bellows murmur, and no second sound of the heart; respiratory sound very indistinct over the lower portion of the lungs; percussion dull in the same locality.

Nov. 5th. I found my patient laboring under still greater dyspnœa than at last visit; pulse more feeble; the sounds of the heart the same; the sibilant râle to be heard over the whole upper portion of the left lung; had been obliged to sit up in his chair all night.

Nov. 6th, 9, A. M. Much more feeble; distress greatly increased; pulse not perceptible at the wrist; died at 2 o'clock, immediately after getting up out of his chair to get a drink of water.

*Post-mortem.* The whole heart much enlarged, but the left ventricle more than any other portion, being much dilated as well as hypertrophied; weight of heart eighteen ounces.

The valves were all healthy except the aortic, which were but two in number, &c.

The diameter of the aortic orifice was also diminished, I think nearly or quite one half.

Effusion of a large quantity of serum in the cavity of the chest, mostly upon the right side; the whole of the lower lobe of the right lung and a part of the lower lobe of left, were carnified; liver congested, large and hard, almost granular.

This patient had never been affected with rheumatism.

*Prof. Flint* exhibited a fine specimen of aneurism of the arch of the aorta. The sac was quite large, and not ruptured.

The patient had been laboring for many years under some affection of the chest.

The tumor has been examined by several physicians prior to his decease. The patient died suddenly, but not from rupture of the aneurism.

The association then adjourned.

AUSTIN W. NICHOLS, M. D.,
Secretary *pro tem.*

